# Faecal biomarkers can distinguish specific mammalian species in modern and past environments

**DOI:** 10.1371/journal.pone.0211119

**Published:** 2019-02-07

**Authors:** Loïc Harrault, Karen Milek, Emilie Jardé, Laurent Jeanneau, Morgane Derrien, David G. Anderson

**Affiliations:** 1 Department of Archaeology, Durham University, Durham, United Kingdom; 2 Department of Archaeology, University of Aberdeen, Aberdeen, United Kingdom; 3 The James Hutton Institute, Craigiebuckler, Aberdeen, United Kingdom; 4 Université Rennes, CNRS, Géosciences Rennes, UMR 6118, Rennes, France; 5 Department of Environment and Energy, Sejong University, Seoul, South Korea; 6 Department of Anthropology, University of Aberdeen, Aberdeen, United Kingdom; University of Illinois, UNITED STATES

## Abstract

Identifying the presence of animals based on faecal deposits in modern and ancient environments is of primary importance to archaeologists, ecologists, forensic scientists, and watershed managers, but it has proven difficult to distinguish faecal material to the species level. Until now, four 5β-stanols have been deployed as faecal biomarkers to distinguish between omnivores and herbivores, but they cannot distinguish between species. Here we present a database of faecal signatures from ten omnivore and herbivore species based on eleven 5β-stanol compounds, which enables us to distinguish for the first time the faecal signatures of a wide range of animals. We validated this fingerprinting method by testing it on modern and ancient soil samples containing known faecal inputs and successfully distinguished the signatures of different omnivores and herbivores.

## Introduction

The signatures of animals in the environment, or on an archaeological site, can be detected by the faecal material they leave behind. Archaeologists, forensic scientists, ecologists, watershed managers and others make use of the organic residues derived from faecal inputs in the environment to determine the presence of animals and/or human activities [1–10] or to pinpoint whether animal faeces were a source of organic nutrients (e.g. in arable soils, [11–18]) or pollutants in catchment basins (e.g. a source of pathogenic bacteria, viruses and protozoa, [19–31]).

Particularly useful are a class of lipids known as 5β-stanols, which are direct biomarkers of animal faeces, and have the important advantage of long-term preservation in soils and sediments due to their low solubility in water and their ability to bind to particulate organic matter [25]. Their distribution in faecal material, called a stanol fingerprint, identifies a particular mammalian species on the basis of its diet (main sterol uptake), its ability to biosynthesize endogenous sterols (secondary sterol uptake) and the way it biohydrogenates sterols and converts them into stanols with the help of digestive tract bacteria (intestinal flora) [19]. The most common techniques used for stanol fingerprinting are currently able to identify whether faeces belong to carnivores, omnivores or herbivores, but do not allow identification of the genus or species. Mainly because of their respective diets, cholesterol-derived 5β-stanols are found in high proportions in the faeces of omnivores and carnivores (coprostanol and epicoprostanol), while 5β-stanols derived from β-sitosterol, a phytosterol (plant sterol), are found in high proportions in herbivore faeces (24-ethylcoprostanol and 24-ethylepicoprostanol) [1]. In order to improve the distinction between the faecal signature of different mammal species in modern [16, 17, 20, 21, 24, 28, 29] and ancient [2, 3, 5, 7, 9, 11–15, 18, 32–34] environments, efforts have been made to develop the technique of faecal steroid biomarker analysis, mainly focused on the ratios of the four above-mentioned important 5β-stanols, sometimes in combination with the analysis of another group of faecal steroids, bile acids. However, the use of ratios calculated from four compounds has significant limitations. Ratio thresholds can and frequently do overlap, making it impossible to distinguish between species [22]. The recent introduction of new 5β-stanol ratios, used in combination with bile acids, has improved the ability to identify some species on the basis of their faeces (e.g. sheep and goats), but even this new method fails in contexts where several species have potentially mingled [10].

A small number of previous studies have applied multivariate analyses of a wider range of steroids, including sterols, 5α- and 5β-stanols and stanones, to deepen the investigation of variations between the faecal fingerprints of different species [4, 19, 23, 26, 27, 31]. However, the sterols and 5α-stanols used in these studies are naturally found in the environment. Sterols and to a lesser extent 5α-stanols may be direct components of soil fauna and vegetation, and 5α-stanols and stanones can also be microbially-mediated degradation products of sterol precursors, which limits their use for faecal fingerprinting [10]. On the other hand, 5β-stanols found in soils almost entirely come from the endogenous biohydrogenation of sterols within the gastrointestinal tract of higher animals, making them useful as biomarkers to identify faecal inputs in soils ([10] and references therein). For faecal fingerprinting with stanols, it is therefore important to restrict the analysis to 5β-stanols alone.

We hypothesized that including the analysis of other 5β-stanol compounds, even though they are present in lower quantities in animal faeces, would significantly improve our ability to derive species-specific faeces fingerprints using multivariate statistics. To test this hypothesis, we analysed the concentrations of eleven faecal stanols in 90 reference samples of animal faeces collected from ten domestic and wild mammalian species (humans, pigs, dogs, cows, horses, goats, sheep, reindeer, lemmings, moose; S1 and S2 Figs, S1 and S2 Tables). We then used multi-variate statistics to develop a faecal fingerprint ‘reference library’. Then, we validated this faecal reference database by testing it on modern and ancient soil contexts with known faecal inputs, where wild and domestic animals and humans intermingled. These were an ethnoarchaeological pastoral site in the Sai͡an Mountains, south-central Siberia, and an archaeological site on the I͡Amal peninsula, north-west Siberia. At the Sai͡an Mountains site, the intention of the lipid study was to confirm and distinguish the presence of *Rangifer* and *Equus* gathering close to a salt lick. At the I͡Amal peninsula site, faecal biomarkers were used to confirm and distinguish the presence of *Rangifer* and *Canine* (and thereby depositing their faecal matter), on the wide flat plain behind the habitation site as partial confirmation of the hypothesis that this site was an early site of reindeer domestication.

Finally, we compared the diagnostic signature obtained with eleven faecal compounds and multivariate statistic models with models including only the four main faecal stanols commonly used in the literature (coprostanol, epicoprostanol, 24-ethylcoprostanol and 24-ethylepicopropstanol) and related diagnostic ratios. This comparison aimed to pinpoint the relevance of including new faecal compounds and diverse statistical tools in studies designed to identify and distinguish between animal signatures at the species level.

## Study areas

### A Tofa hunting camp in the Sai͡an Mountains

Tofas are an indigenous people living in southern Siberia within that portion of the Sai͡an Mountains that intersects with Irkutsk oblast’. Traditionally, they hunt ungulates and fur-bearers in the forests with the use of a variety of domestic animals including horses, domestic reindeer and dogs [35]. Tofa multi-species forest adaptations are considered a “classic case” in models of the origin of animal domestication in Eurasia. Several authors, as far back as the 19^th^ century, have identified Tofa pastoralism as a possible origin point of animal domestication in Eurasia [36–39].

The test site was a winter hunting camp in the eastern Sai͡an Mountains on the upper Dugul’ma River (N 53°27.158’, E 098°38.985’) at an elevation of 1458 m. In use since 2000, it consisted of a clearing located on two stepped alluvial terraces, surrounded by dense taiga forest. On the lower alluvial terrace, there was a furnished wooden cabin built for the winter hunting season. The clearing was used for making outdoor cooking fires and congregating reindeer and horses, and there was a wooden shelter for dogs. There were a couple of boulders used as salt licks for reindeer and horses, and the area around these boulders was heavily trampled. On the upper alluvial terrace there was a foundation for a round tent (including the tent poles) used every spring and autumn by the mobile herders who monitor the domestic reindeer herds for the nearby village of Alygdzher.

Hunters based in Alygdzher come to this hunting camp on horseback every autumn and use it as a base camp while they fetch their riding reindeer (male castrates) from their lichen-rich autumn pastures at higher elevations, in preparation for the winter hunting season. This takes each hunter a number of days/weeks, and anywhere between two and six hunters may use the cabin at any one time. When the riding reindeer have been caught, they are brought back to the camp and tied to trees and cut logs on the edges of the clearing. They are taken back up to lichen-rich pastures every day for a couple of hours of grazing, but otherwise remain at the camp until their owner has finished fetching the 15–25 reindeer he will use for the winter hunting season. Occasionally the reproductive reindeer herd (females, calves, and a reproductive bull) also moves through the clearing, attracted by the salt put out by the hunters. While the hunters are engaged with finding and feeding reindeer, their horses are hobbled and left to free-range forage, but they do not stray far, and frequently come back to the clearing for salt. Thus, for approximately two to three weeks every autumn, humans, horses, reindeer, and dogs co-mingle at the site.

### An archaeological site on the I͡Amal peninsula

The archaeological site known as I͡Arte 6 is located at co-ordinates 68°54'21.3" N, 69°57'36.8" E on a terrace 20m above the I͡Uribeĭ River, within the I͡Amal county of the I͡Amalo-Nenet͡s Autonomous District of Ti͡umen' oblast’, Russian Federation. The site was first documented in 1988 by an archaeological expedition led by the Tobol’sk State Pedagogical Institute and has since been excavated six times by a number of Russian and international teams between 1992 and 2015 [40–43]. It forms part of a chain of habitation sites along the river associated with the Tiuteĭ Sale archaeological culture, distinguished by its ceramics. Initial dendrochronological dates put the time of occupation at the end of the 11^th^ century AD (1071–1106) [44]. The I͡Arte 6 site was distinguished by the foundations of semi-subterranean dwellings visible at the surface, a significant ditch interpreted at the time as a defensive structure, and significantly deep and rich cultural layers made up from the accumulation of over 30,000 animal bones and bone fragments, the majority of which came from migratory and/or domestic reindeer (*Rangifer tarandus*). Other significant species represented were Arctic fox (*Vulpus*), birds, and a number of dog and/or wolf skeletons. Among the artefacts found well-preserved in permafrost were large collections of bone tools, many of which were designed for the working of *Rangifer* skins, and a collection of halters, swivels, and buckles interpreted, by analogy, to the gear used to harness domesticated reindeer and dogs today. The artefacts at this site, along with those at three others in the region, were prominently advertised by Natal’i͡a Fedorova as evidence of a far Northern origin point for domesticated reindeer husbandry [45, 46].

The entire terrace is still intensively used today by Nenets reindeer herders. Today’s herders often camp for several weeks on the south bank of the I͡Uribeĭ River in the early spring, if they cannot cross on ice, and wait for the ice to break and the river to subside before crossing to move to their summer pastures on the far north of the peninsula. Nenets folklore associates the site with a former encampment of a previous metal-working nomadic people known as Sikhirti͡a, who also harnessed domesticated animals and worked with dogs [47].

The dominant archaeological interpretation of the site is that it was a seasonal hunting camp for slaughtering and processing migratory wild *Rangifer*, which once frequented the peninsula [43]. By contemporary analogy to modern Nenetses, it is thought that around 20 people stayed at the site seasonally, and that they were supported by up to 250 head of domesticated reindeer and a small number of domesticated dogs [42].

## Materials and methods

### Reference sample collection

Ninety faecal samples from ten different species were collected for this study and other environmental research projects. Most of these samples were collected directly from the ground and represented a composite sample of several individuals of the same species (S2 Table). Wastewater samples from wastewater treatment plants (WWTP) were sampled and considered as human faecal samples. Faecal samples originating from Scotland and France were freeze-dried before pre-treatment and lipid extraction. Samples originating from Fennoscandia and Russia were air-dried before pre-treatment and lipid extraction due the lack of freeze-drying facilities while conducting remote fieldwork. Air-drying was conducted by putting samples in aluminium trays and letting them dry in a field laboratory tent over several days, during which temperatures ranged between 5 and 15°C during the night and from 10–30°C during the day. The field studies did not involve any protected species. Permission is not required to sample gather faeces for reference samples from domestic or wild animals in the Russian Federation.

### Soil sampling

At the Tofa hunting camp, seven soil samples were collected at a 5m- interval on a 30m-long east-west transect through a part of the camp used frequently by horses and reindeer attracted by a salt lick. The soils were sampled during the autumn of 2014 by excavating a 10x10 cm hole and removing the top 3 cm of the surface soil, producing a soil sample of around 100 g. At the Nenets site, we systematically mapped the soils next to the site on a 5–10 m grid using 0.5x0.5 m test pits. Two to four buried soils were found in each test pit, which had been buried by layers of windblown sand. 100 g soil samples were taken for 5β-stanol analysis, and charcoal found in the buried soils was radiocarbon dated to confirm the contemporaneity of the soils with the I͡Arte 6 site (S2 and S3 Tables).

For both sites, once sampled, soils were air-dried as previously described for the faecal reference samples due to the lack of freeze-drying facilities in the field. The soil from the plain behind the archaeological site I͡Arte 6 was samples under Discover Licence (oktrytyĭ list) No.647 of 19 June 2015 from the Ministry of Culture of the Russian Federation within the excavation organized by Andreĭ Vladimirovich Plekhanov. The soil survey at the Sai͡an site was conducted with the permission of the lead Tofa hunter and herder to which the hunting camp was registered.

### Dating method

Charred wood was recovered from dried and sieved (2mm) soil samples taken from four buried soil horizons adjacent to the I͡Arte 6 site (see S2 and S3 Tables). The wood was identified as Salix and Betula roundwood, suitable for radiocarbon dating. AMS radiocarbon assays were conducted by the Poznań Radiocarbon Laboratory, Poland, and were calibrated using OxCal 4.2.4 [48, 49] using the IntCal 13 calibration atmospheric curve [50].

### 5β-stanol analysis

Faeces and soil samples were analysed according to four different methods. The method used for each sample can be found in S2 Table. All solvents used were HPLC-grade.

The ASE-SIM-QP2010 method was performed following the published method [22]. Briefly, lipids of freeze-dried samples were extracted with dichloromethane (DCM) using an Accelerated Solvent Extractor (ASE 200, Dionex). Lipids were fractionized by solid-liquid chromatography to isolate polar compounds, containing 5β-stanols. The polar fraction was derivatized with a mixture of N,O-bis-(trimethylsilyl)trifluoroacetamide and trimethylchlorosilane (BSTFA + TMCS, 99/1, v/v, Supelco) after addition of 5α-cholestane (CDN isotope) as an internal standard (IS). Derivatized samples were analysed by a combined gas chromatograph-mass spectrometer (GC-MS), Shimadzu QP2010plus. The capillary column used was 60 m-long with an inner diameter of 0.25 mm (SLB-5ms, Supelco). Analyses were carried out in selective ion monitoring (SIM) mode (main fragments can be found in S1 Table). Identification of compounds was made by retention time and mass spectra comparison with those of available standards or data in the literature (S1 and S2 Figs, S1 Table). Quantification was achieved with 5-point internal calibration curves of available standards with relevant fragments, and comparison with a constant IS concentration added prior to analysis. The limit of quantification was 30 ppb.

The SPE-SIM-QP2010 method was performed on waste water treatment plant effluents (for the human reference samples) as previously described [24]. Briefly, lipids from filtered samples were extracted by solid phase extraction (SPE) through ENVY disks (Supelco) and analysed and quantified by GC-MS according to the ASE-SIM-QP2010 method. The limit of quantification was 30 ppb of water sample.

The sonication-SIM-QP2010 method is adapted from previous methods [26, 51, 52]. Soil and faecal samples were dried, crushed and then 1-mm sieved. 2 g of soil sample or 0.2 g of faecal sample were put in 20 ml Pyrex centrifugation tubes, then a known amount of 5β-cholan-24-ol (Chiron) was added as a recovery standard (0.5 μg for soils samples and 10 μg for faecal samples). Lipids were extracted in an ultrasonic bath at 30°C for 15 min with 15 ml of a DCM/MeOH mixture (2/1, v/v). Suspensions were centrifuged at 1500 rpm and 10°C for 10 min and filtered in glass columns through DCM-washed and packed cotton wool. Extractions were repeated two times and the three extracts pooled together. The volume of pooled extracts was reduced under a slight nitrogen stream at 40°C before further centrifugation at 3500 rpm and 10°C for 10 min. Then, suspensions were filtered in glass columns through DCM-washed and packed cotton wool and nitrogen-dried. Dried lipid extracts were saponified in 20 ml Pyrex tubes with 1.5 ml of a 1 M KOH/MeOH mixture (KOH from Sigma) at 90°C overnight (ca. 14 h). Saponified extracts were transferred into a 10 ml separatory funnel for liquid-liquid extraction. 1.5 ml of deionised water were added and extractions were performed with 3 x 2 ml of DCM, then the neutral fractions collected were pooled. Residual water was removed from neutral fractions by filtration through glass columns packed with anhydrous sodium sulphate Na_2_SO_4_ (Sigma). Neutral fractions were nitrogen-dried and re-dissolved in *n*-heptane. Neutral fractions were separated into apolar and polar (containing 5β-stanols) fractions by solid-liquid chromatography in glass columns with silica gel (in *n*-heptane). Apolar fractions were eluted with 3 x 2 ml of *n*-heptane and 3 x 2 ml of a *n*-heptane/DCM mixture (2/1, v/v), then polar fractions were eluted with 4 x 1 ml of a DCM/MeOH mixture (2/1, v/v). Then, polar fractions were nitrogen-dried and re-dissolved in DCM. Except for the GC oven temperature program, derivatization and analyses of polar fractions were carried by GC-MS as described in the ASE-SIM-QP2010 method. GC oven temperature program started at 80°C for 1.5 min, then increased to 275°C at 12°C min^-1^, then increased to 300°C at 0.8°C min^-1^, then increased to 320°C at 10°C min^-1^ held for 25 min. Analyses were carried in SIM mode (main fragments can be found in S1 Table). Quantification was achieved with 5-point internal calibration curves of available standards with relevant fragments, and comparison with constant IS concentration added prior to analysis. The limit of quantification was 10 ppb.

The saponification-TIC-TRACE DSQ method was performed as previously described [20]. Briefly, 5β-cholan-24-ol (Chiron) was added to ca. 0.1 g of dried sample as IS. Both lipid extraction and hydrolysis of ester functions were achieved in the meantime by saponification with ethanolic KOH (VWR). The fraction containing 5β-stanols was isolated by successive liquid-liquid extraction and solid-liquid chromatography. After derivatization with a mixture of BSTFA-pyridine, 5β-stanols were analysed by GC-MS on a Trace GC (Thermo Fisher Scientific) equipped with a ZB-5HT capillary column (Phenomenex, 30 m × 0.25 mm ID, 0.25 μm film thickness) coupled to a Trace DSQ MS (Thermo Fisher Scientific) running in full scan mode. Quantification was achieved by comparison of targeted compound total ion current (TIC) area with those of the IS added before lipid extraction. The limit of quantification was 100 ppb.

### Statistical analysis

**Fig 1:**
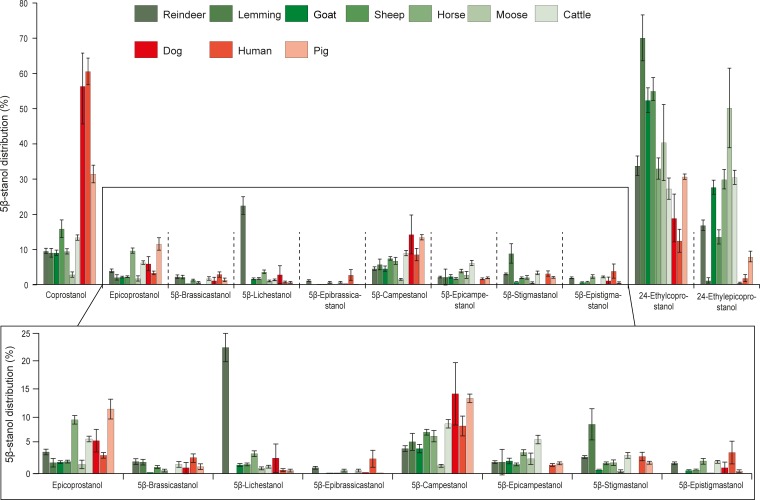
Distributions of 5β-stanols in herbivore and omnivore faecal material. Compound information can be found in S1 Table and S2 Fig. Mean ± SE. Individuals: n(reindeer) = 23, n(lemming) = 6, n(goat) = 9, n(sheep) = 12, n(horse) = 7, n(moose) = 5, n(cattle) = 9, n(dog) = 4, n(human) = 8, n(pig) = 7. Individual sample information and 5β-stanol distributions can be found in S2 Table.

**Fig 2:**
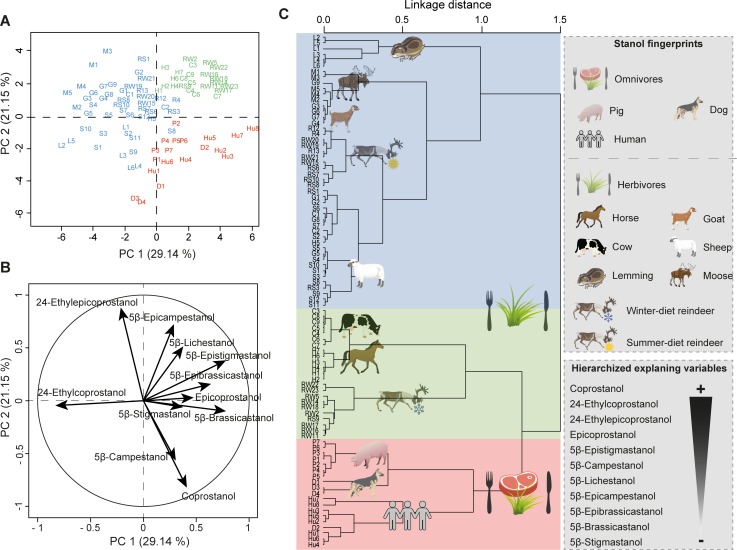
Mammal fingerprints. (**A**) PCA score plot of 5β-stanol distributions in reindeer (R), lemming (L), goat (G), sheep (S), horse (H), moose (M), cattle (C), pig (P), dog (D) and human (Hu) faecal samples. Colours represent the main clusters obtained by HCPC (**C**). PC 1 and PC 2 represent respectively principal components 1 and 2; numbers in brackets show the variance explained by each PC. (**B**) PCA correlation circle. (**C)** HCPC dendrogram of all species fingerprints built on PCs from the PCA. The main variables/compounds responsible for the distinction between the main clusters are hierarchized (from the more important + to the less import -) according to S4 Table. More details of the PCA/HCPC models can be found in S4 Table and sample information is in S2 Table.

To compare data generated from different methods and to compare faeces to soil samples, 5β-stanol concentrations were transformed into their relative abundance (%) compared to their sum (Fig 1). Prior to statistical analyses, 5β-stanol relative abundances were arcsine (√ %)-transformed to normalize distributions and increase homoscedasticity as previously recommended [53]. Multivariate analyses were performed with open-source R [54] and RStudio Desktop [55] software, using the FactoMineR package and the related Rcmdr graphical interface [56]. To investigate the differences between 5β-stanol fingerprints in herbivore faecal samples, principal component analysis (PCA) was performed on the transformed relative abundance of each compound (or variable, S4 Table) followed by a hierarchical clustering on principal components (HCPC, Fig 2A–2C) using Euclidian distances and Ward’s method.

**Fig 3:**
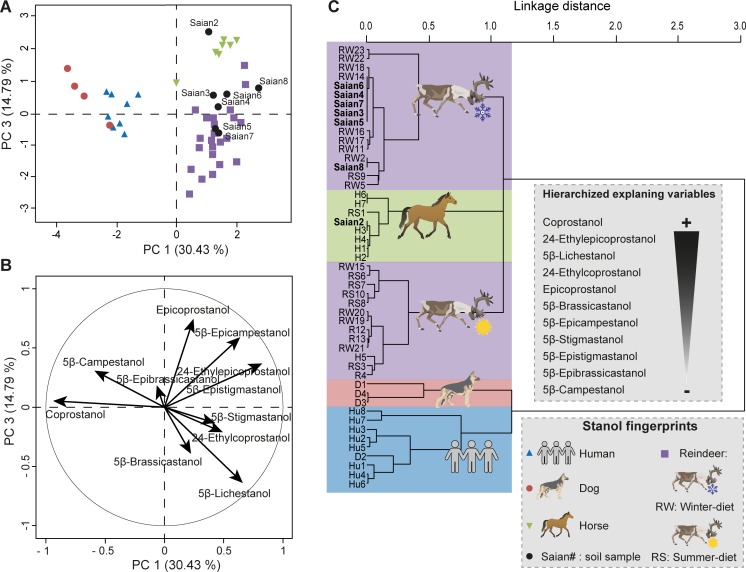
Identification of soil faecal fingerprints from the contemporary Tofa (Sai͡an Mountains) camp by multivariate analyses on eleven 5β-stanol distributions. (**A**) PCA score plot of 5β-stanol distributions from the reference library of faeces of dogs, horses, humans and reindeer, together with soil samples from the site. PC 1 and PC 3 represent principal components 1 and 3 respectively. The numbers in brackets show the variance explained by each PC. (**B**) PCA correlation circle. (**C**) HCPC dendrogram of dog (D), horse (H), human (Hu), reindeer (R) and soil sample (Saian) fingerprints. The main variables/compounds responsible for the distinction between the main clusters are hierarchized (from the more important + to the less import -) according to S5 Table. More details of the PCA/HCPC models can be found in S5 Table and sample information is in S2 Table.

This fingerprinting model was tested on the modern Sai͡an Mountain site where known species used to be and are present. To do so, predictive PCA and HCPC were performed with the transformed 5β-stanol distributions of dog, horse, human and reindeer faecal samples as other domestic species from our fingerprint library (cows, pigs, goats, and sheep) were not present in the study area, and were therefore excluded from the PCA and HCPC models (Fig 3A–3C, S5 Table). To determine whether a soil sample had a fingerprint similar to one of the species present, their 5β-stanol distribution was added to the previous predictive PCA and HCPC models as supplementary individuals. To do so, faecal samples were treated as active variables by weighting their transformed 5β-stanol distributions with 1, while soil samples were treated as supplementary individuals by weighting their transformed 5β-stanol distributions with 10^−20^.

**Fig 4:**
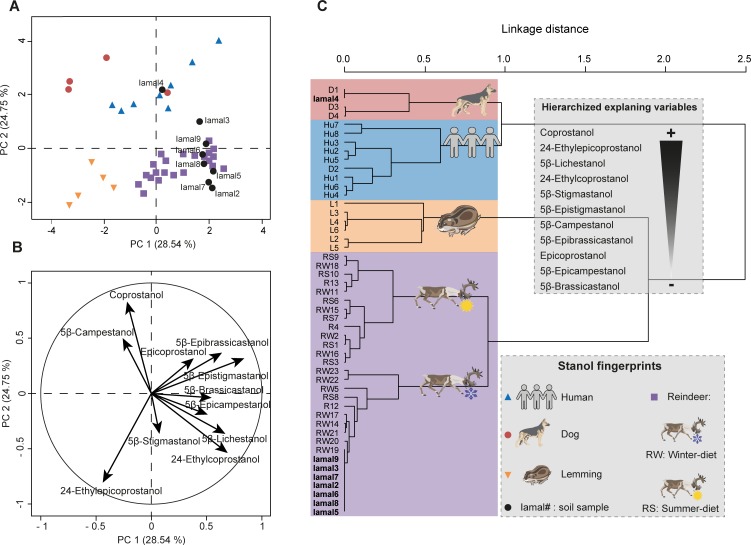
Identification of soil faecal fingerprints from the archaeological site of I͡Arte VI by multivariate analyses on eleven 5β-stanol distributions. (**A**) PCA score plot of 5β-stanol distributions from the reference library of faeces of dogs, lemmings, humans and reindeer, together with the 5β-stanol distributions in the soil samples. PC1 and PC2 represent respectively principal components 1 and 2. The numbers in brackets show the variance explained by each PC. (**B**) PCA correlation circle. (**C**) HCPC dendrogram of dog (D), lemming (L), human (Hu), reindeer (R) and soil sample (Iamal) fingerprints. The main variables/compounds responsible for the distinction between the main clusters are hierarchized (from the more important + to the less import -) according to S6 Table. More details of the PCA/HCPC models can be found in S6 Table and sample information is in S2 Table.

This fingerprint methodology was then applied to the I͡Amal peninsula site context, with the exception that faecal reference fingerprints from horses, cows, pigs, sheep, and goats, species which one would not expect to find in high-latitude tundra environment, were removed from the predictive PCA model and HCPC. The faecal signatures of transitory Arctic fox and birds were excluded from the model, since people likely brought them to the site as carcasses, and their faecal input in the soils was likely to be insignificant or non-existent. As a consequence, according to the site context (see Study areas section), dog, human, reindeer and wild lemming (potential input of faecal material in Siberian soils) were the four species considered in the site PCA/HCPC models (Fig 4A–4C and S6 Table).

In both of our case studies, soil samples were judged to have faecal material present when the ratio of the sum of 5β-stanols compared to those collected from an off-site control sample was greater than 10 (S2 Table). The samples that satisfied this faecal stanol concentration requirement were then analysed using PCA and HCPC to determine their 5β-stanol fingerprint and the species-specific sources of the faecal input in the soil were determined by comparison with the reference library of faecal fingerprints.

**Fig 5:**
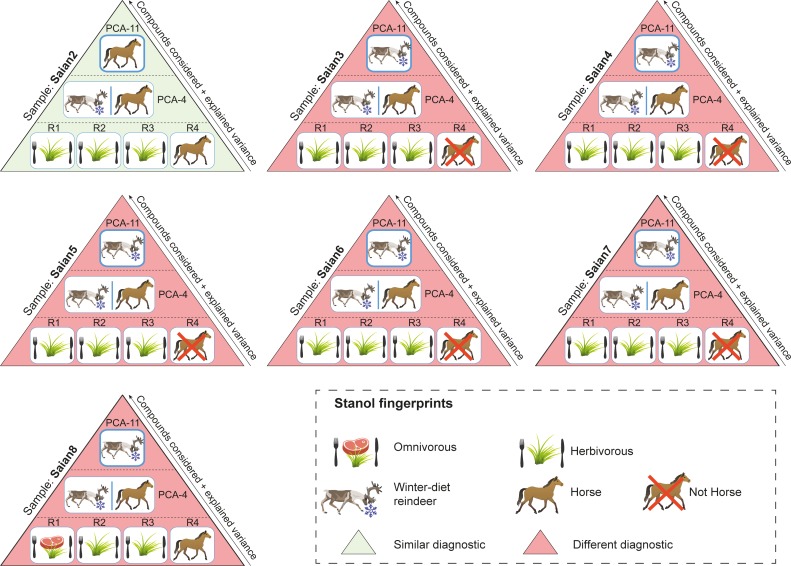
Summary comparison of diet and species identification using ratios and multivariate analyses for soil samples taken from the Tofa site (Sai͡an Mountains). More details are presented in S7 Table. R1 refers to the ratio of distributions of coprostanol/(coprostanol + 24-ethylcoprostanol) used to discriminate the herbivore fingerprint from human (herbivore = 0.38 < R1 < 0.73 = human, [20]). R2 refers to the ratio of distributions of (coprostanol + epicoprostanol)/(24-ethylcoprostanol + 24-ethylepicoprostanol) used to identify the omnivore fingerprint (R2 > 1, [7]). R3 refers to the ratio of distributions of epicoprostanol/(cholestanol + coprostanol) used to discriminate the herbivore fingerprint from human (human = 0.01 < R3 < 0.1 = cattle and horse, [21]). R4 refers to the ratio of distributions of (24-ethylepicoprostanol/24-ethylcoprostanol) + (epicoprostanol/coprostanol) used to discriminate the horse fingerprint from other herbivores (No horse = 0.8 < R4 < 1.2 = horse, [10]). PCA-4 refers to the predictive PCA and its corresponding HCPC built with the distribution of the four main 5β-stanols (coprostanol, epicoprostanol, 24-ethylcoprostanol and 24-ethylepicoprostanol) in the human, dog, horse and reindeer reference samples from our database. PCA-11 refers to the predictive PCA and its corresponding HCPC built with the distribution of eleven 5β-stanols in the human, dog, horse and reindeer reference samples from our database. Reindeer and horses are herbivores so diagnostics between ratios and multivariate analyses can be compared.

**Fig 6:**
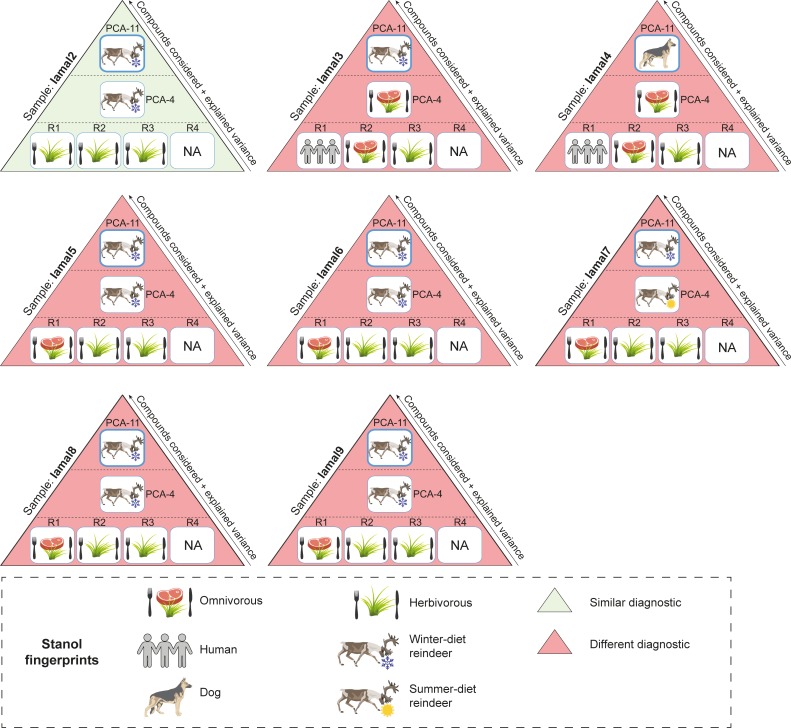
Summary comparison of diet and species identification using ratios and multivariate analyses for I͡Amal soil samples. More details are presented in S7 Table. R1 refers to the ratio of distributions of coprostanol/(coprostanol + 24-ethylcoprostanol) used to discriminate the herbivore fingerprint from human (herbivore = 0.38 < R1 < 0.73 = human, [20]). R2 refers to the ratio of distributions of (coprostanol + epicoprostanol)/(24-ethylcoprostanol + 24-ethylepicoprostanol) used to identify the omnivore fingerprint (R2 > 1, [7]). R3 refers to the ratio of distributions of epicoprostanol/(cholestanol + coprostanol) used to discriminate the herbivore fingerprint from human (human = 0.01 < R3 < 0.1 = cattle and horse, [21]). R4, the diagnostic ratio for the horse fingerprint, is “not applicable” (NA) because no horses were present on this high Arctic site (see Text). PCA-4 refers to the predictive PCA and its corresponding HCPC built with the distribution of the four main 5β-stanols (coprostanol, epicoprostanol, 24-ethylcoprostanol and 24-ethylepicoprostanol) in the human, dog, lemming and reindeer reference samples from our database. PCA-11 refers to the predictive PCA and its corresponding HCPC built with the distribution of eleven 5β-stanols in the human, dog, lemming and reindeer reference samples from our database. Reindeer and horses are herbivores so diagnostics between ratios and multivariate analyses can be compared.

Finally, we tested the relevance of our fingerprint method using eleven faecal stanols by comparing soil sample fingerprints from the two sites identified with eleven 5β-stanols to those identified with PCA/HCPC models based on only the four main 5β-stanols commonly used in the literature (coprostanol, epicoprostanol, 24-ethylcoprostanol and 24-ethylepicoprostanol) and also four stanol ratios used in the literature as diet or species identification index (Figs 5 and 6, S7 Table):

R1 = Coprostanol / (Coprostanol + 24-Ethylcoprostanol); with herbivore = 0.38 < R1 < 0.73 = human [20]
R2 = (Coprostanol + Epicoprostanol) / (24-Ethylcoprostanol + 24-Ethylepicoprostanol); with omnivore > 1 [7]
R3 = Epicoprostanol / (Cholestanol + Coprostanol); with human = 0.01 < R3 < 0.1 = cattle and horse [21]
R4 = (24-Ethylepicoprostanol / 24-Ethylcoprostanol) + (Epicoprostanol / Coprostanol); with no horse = 0.8 < R4 < 1.2 = horse [10]

**Fig 7:**
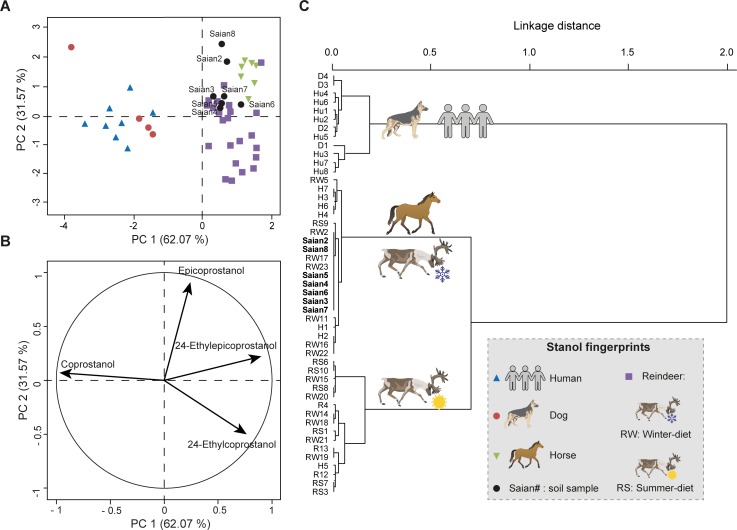
Multivariate analyses to identify the faecal fingerprints of soil samples from the Tofa site (Sai͡an Mountains) based on only the four compounds commonly used in the literature: coprostanol, epicoprostanol, 24-ethylcoprostanol and 24-ethylepicoprostanol. PCA and HCPC of the four main 5β-stanols distribution (arcsine-root transformed) of reference faecal material from dog (D), horse (H), human (Hu) and reindeer (R) and soil samples (Saian). Reference and soil samples nomenclature can be found in S2 Table. (**A**) PCA score plot of PC1 and PC2. The high variance explained by the first two PCs is due to the low number of variables (four). (**B**) PCA correlation circle of PC1 and PC2. (**C**) Dendrogram obtained by HCPC from the first two PCs of the PCA.

**Fig 8:**
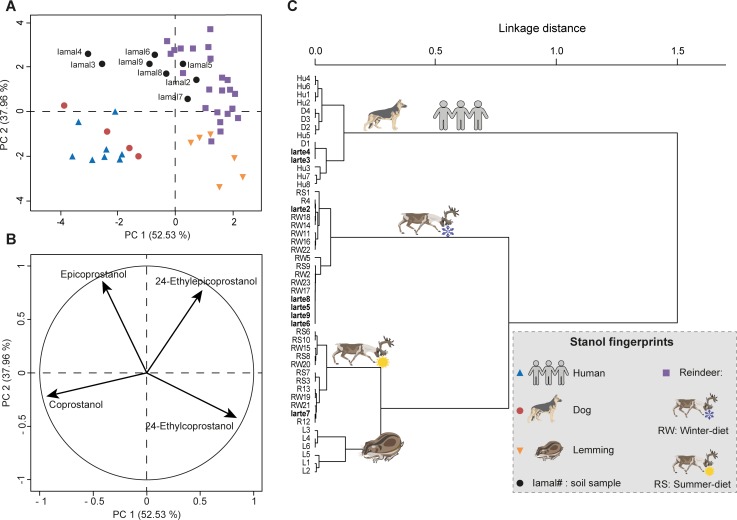
Multivariate analyses to identify the faecal fingerprints of soil samples from the I͡Amal peninsula site based on only the four compounds commonly used in the literature: coprostanol, epicoprostanol, 24-ethylcoprostanol and 24-ethylepicoprostanol. PCA and HCPC of the four main 5β-stanol distribution (arcsine-root transformed) of reference faecal material from dog (D), human (Hu), lemming (L) and reindeer (R) and soil samples. Reference and soil sample nomenclature can be found in S2 Table. (**A**) PCA score plot of PC1 and PC2. The high variance explained by the first two PCs is due to the low number of variables (four). (**B**) PCA correlation circle of PC1 and PC2. (**C**) Dendrogram obtained by HCPC calculated from the first two PCs of the PCA.

The four-compound PCA/HCPC models were run with the arcsine (√ %)-transformed relative abundances of these four compounds (sum = 100%) for the relevant species for each site (Sai͡an site: Fig 7A–7C, S8 Table; and I͡Amal site: Fig 8A–8C, S9 Table).

## Results and discussion

### Diet and species fingerprints

#### Omnivore versus herbivore fingerprints

The analysis of 90 faecal samples from 10 mammal species allowed us to identify and quantify eleven 5β-stanols (Fig 1, S2 Table), in cases when usually no more than six compounds were detected [4, 19, 22, 31, 34]. On average for all the species studied here, the five main 5β-stanols in faeces were 24-ethylcoprostanol, 24-ethylepicoprostanol, coprostanol, epicoprostanol and 5β-campestanol. Other compounds, 5β-epicampestanol, 5β-stigmastanol and 5β-epistigmastanol accounted for less than 5% of the distribution. In addition, three previously unreported compounds were present in noticeable quantities: 5β-lichestanol (S2A Fig), 5β-brassicastanol and 5β-epibrassicastanol (S2B Fig). On average, 5β-lichestanol, believed to come from lichesterol provided by a diet based on lichen [57], represented around 22% of the total 5β-stanols in reindeer faecal samples and less than 2% in other species faecal samples, which do not feed on lichesterol-rich lichen (Fig 1, S2 Table). 5β-Brassicastanol constituted on average less than 2% of 5β-stanols for all species and 5β-epibrassicastanol less than 1% (Fig 1, S2 Table).

According to previous findings [1, 19, 23, 33], the faeces of omnivores (humans, pigs and dogs) contained a higher relative abundance of coprostanol compared to herbivores (cattle, horses, goats, sheep, reindeer, lemmings, moose), which showed a high relative abundance of both 24-ethylcoprostanol and 24-ethylepicoprostanol (Fig 1). This trend is confirmed when calculating the commonly used R1 ratio (coprostanol / (coprostanol + 24-ethylcoprostanol); herbivore = 0.38 < R1 < 0.73 = human, [20]) and R2 ratio ((coprostanol + epicoprostanol) / (24-ethylcoprostanol + 24-ethylepicoprostanol); omnivore > 1, [7]) of our faecal samples (S2 Table). This omnivore/herbivore fingerprint distinction, mainly driven by coprostanol, epicoprostanol, 24-ethylcoprostanol and 24-ethylepicoprostanol distribution, is also confirmed by the numerical outputs of the HCPC model run on the eleven 5β-stanol distribution for all 10 species (Fig 2C, S4 Table): the four main explaining variables used to build the hierarchical tree and clustering species fingerprint are coprostanol > 24-ethylcoprostanol > epicoprostanol > 24-ethylepicoprostanol and then the remaining compounds to a lesser extent. This is in agreement with Derrien et al. [22], who found that the main compounds allowing the faecal distinction between omnivores (pigs and humans) and omnivores (cattle) in their PCA model were coprostanol and 24-ethylcoprostanol (and sitostanol). The same trend was noticed by Leeming et al. [19] whose pioneering PCA model, built on the concentration of steroidal compounds (including sterols, stanones, 5α- and 5β-stanols) from various species, made it possible to distinguish omnivore signatures (mainly humans and pigs) from herbivore signatures (mainly cattle, sheep and horses). In their model, omnivore faeces contained high concentrations of C_27_ steroidal compounds (i.e. cholesterol and its transformation products 5α-cholestanol, coprostanol, epicoprostanol etc.), while herbivore faeces were dominated by C_29_ compounds (i.e. sitosterol and its transformation products 5α-sitostanol, 24-ethylcoprostanol, 24-ethylepicoprostanol etc.). In a similar study, Shah et al. [4] were able to distinguish a human faecal fingerprint from omnivores (dogs and dingos), herbivores (pig, lamb, cow, donkey, horse, water buffalo, rabbit, kangaroo, koala) and birds (chicken, duck and turkey) using multivariate analyses (hierarchical clustering and canonical analysis) based on the concentration of steroidal compounds in animal faeces (cholesterol, campesterol, stigmasterol, sitosterol, 5α-cholestanol, coprostanol, epicoprostanol and 24-ethylcoprostanol). In their model, human and carnivore fingerprints were closely linked relative to herbivore fingerprints, which were more similar to that of birds. This is most probably because of the higher concentration of cholesterol (main zoosterol) and coprostanol in human and carnivore faeces compared to herbivore and bird ones.

In agreement with the literature, our findings confirm that the distribution of the four main 5β-stanols derived from the main zoosterols and phytosterols (i.e. cholesterol-derived coprostanol and epicoprostanol, and sitosterol-derived 24-ethylcoprostanol, 24-ethylepicoprostanol) in faeces provide sufficient information to distinguish human/omnivore fingerprints from herbivore fingerprints. Nevertheless, these four compounds, as well as non-faecal compounds (sterols and 5α-stanols) may be inadequate to distinguish faecal fingerprints at the species level within a diet group (omnivore, carnivore and/or herbivore).

### Omnivore species fingerprints

Among omnivores, the distinction between species, or at least between humans and others (pigs and dogs), has already been studied using stanol ratios [7, 10, 20, 21, 33, 58] or with multivariate analyses [4, 19, 23, 24, 27, 28, 31].

According to these studies, there is a clear distinction between human and pig faecal fingerprints, which is mainly explained by their differences in coprostanol, epicoprostanol, 24-ethylcoprostanol and 24-ethylepicoprostanol (Figs 1 and 2C, S4 Table).

We found that dog faeces contained a large amount of coprostanol (ca. 56%) associated with a significant amount of 24-ethylcoprostanol (ca. 19%) and 5β-campestanol (Fig 1, S2 Table). Dog faecal lipid biomarkers have only rarely been studied previously, because they are not of prime importance in water management, or the fertilization of ancient agricultural soils. The two studies in which dog faecal biomarkers were analysed had opposite results. While Leeming et al. [19] found mainly coprostanol (5β-stanols considered: coprostanol, epicoprostanol, 5β-stigmastanol, 5β-epistigmastanol, 24-ethylcoprostanol, 24-ethylepicoprostanol), Shah et al. [4] found that coprostanol represented only ca. 1% of the total 5β-stanols (coprostanol, epicoprostanol (0%) and 24-ethylcoprostanol (99%). Diet being one of the main factors explaining faecal biomarker distribution in faeces [19], the differences observed here and in Leeming’s study are likely to be due to the different diets of the dogs studied. This kind of plasticity is illustrated in Fig 2C, where the faecal dog sample D2 is in the same cluster as human faecal samples. Compared to the three other dog faecal samples, which were sampled from non-remote Scandinavian areas, this sample was collected from a dog owned by a Nenets family living far in Northern Siberia, and Nenets people are known to roughly share the same diet as their dogs–who are fed scraps of fish and meat not consumed by humans [59]. Having a similar diet to humans, even without the same metabolism and intestinal flora, it is not surprising that this dog had faeces rich in coprostanol, as humans do. Another important factor for the faecal biomarker distribution in faeces [19], the composition of the intestinal flora, might also explain the different results observed here and in Leeming et al. [19], as microbial gut communities can differ among dog breeds [59, 60]. Despite the poor ability of dogs to convert cholesterol to faecal stanols (mainly coprostanol, [19]) compared to humans (S2 Table), their faecal stanol fingerprint is specific enough to be distinguished from those of humans and pigs (Fig 2C). Interestingly, the distribution of the four main 5β-stanols (coprostanol, epicoprostanol, 24-ethylcoprostanol and 24-ethylepicoprostanol) in dog faeces is quite similar to those of humans, which highlights the importance of the other 5β-stanols (e.g. 5β-epibrassicastanol, 5β-epicampestanol and 5β-stigmastanol; Fig 1) to distinguish between the faeces of these two species, even if they are present in lower concentrations.

### Herbivore species fingerprints

Among herbivore species, we found large differences in the relative abundance of various 5β-stanols, dominated by 24-ethylcoprostanol and 24-ethylepicoprostanol (Fig 1), as expected from phytosterol-based diets (mainly sitosterol). Thanks to multivariate analyses (PCA and HCPC), the distinctive distributions of 5β-stanols of the seven different herbivore species observed in Fig 1 made it possible to distinguish between several herbivore species’ fingerprints (Fig 2A–2C, S4 Table). The analysis showed two main fingerprint groups distinguished on principal component 1 (PC1, Fig 2A and 2C) mainly by the relative abundances of 24-ethylcoprostanol, 5β-epistigmastanol, 5β-brassicastanol and 5β-epibrassicastanol (Fig 2B, S4 Table): one group with horses, cattle and winter-diet reindeer (lichen-based diet), and a second one with lemmings, sheep, moose, goats and summer-diet reindeer (i.e. a more diverse diet based on lichen, grass and shrubs).

Within the first group, winter-diet reindeer fingerprints were well separated from horse and cow mainly by the PC3 (mainly 5β-lichestanol, epicoprostanol and 5β-brassicastanol (Fig 2C, S4 Table). Previous studies unsuccessfully tried to identify reindeer-specific faecal lipid biomarkers due to their unique lichen-rich diet during winter [61], but reindeer faeces were never analysed for faecal stanol biomarkers so comparable data are lacking. Nevertheless the high 5β-lichestanol content found in reindeer faeces, most probably derived from their lichen-rich diet, especially during winter [62], is the main variable allowing such a distinct fingerprint (Fig 1, S2 Table).

Horse and cow fingerprints were separated well into two distinctive sub-clusters, mainly by epicoprostanol, 5β-lichestanol and 5β-campestanol (Fig 1, S4 Table). Interestingly, even if the number of 5β-stanols considered here is different from the study of Gill et al. [34] (coprostanol, epicoprostanol, 5β-campestanol, 5β-epicampestanol, 24-ethylcoprostanol and 24-ethylepicoprostanol), the trends observed in the distribution of faecal stanols from cows and horses are similar. On the contrary, Leeming et al. [19] observed different trends compared to ours when considering six faecal stanols (coprostanol, epicoprostanol, 5β-stigmastanol, 5β-epistigmastanol, 24-ethylcoprostanol and 24-ethylepicoprostanol), but these differences might be due to the fact that they did not find any 24-ethylepicoprostanol in cow and horse faeces.

Within the second group, the faecal fingerprints of lemmings were well separated from those of sheep, summer-diet reindeer, goats and moose (Fig 2C). This distinction is mainly due to the near absence of 24-ethylepicoprostanol in lemming faeces (Fig 1). Within the cluster comprising sheep, summer-diet reindeer, goats and moose, the species fingerprints were less distinguished. Nevertheless, summer-diet reindeer and sheep fingerprints were well separated from other species, in the case of reindeer faeces because of their higher 5β-lichestanol content, and in the case of sheep faeces because of their higher epicoprostanol content (Fig 1). In this ten-species HCPC model including seven herbivores, goats have the least specific fingerprint and individual fingerprints are clustered together with both moose and sheep. This lack of specificity could be the result of different factors. Firstly, goats are known to graze on a greater variety of plants compared to other herbivores [63] and as diet is a key factor in faecal stanol fingerprinting [19], the different diets inherent in different goat samples could partly explain their heterogeneity [64]. Secondly, from a purely statistical point of view, this HCPC model and inherent clustering was built to maximize the explained variance between ten species, including omnivores with a very different stanol fingerprint compared to those of herbivores, and not to maximize the explained variance between particular species relevant to a specific context (i.e. including less species fingerprints).

This lack of specificity of the PCA model to distinguish species fingerprints at a certain level highlights the importance of pre-screening when trying to apply this database to specific case studies in order to narrow the database used to only the species that have the potential to be present on a site [10]. For ecologists, watershed managers, and soil forensic scientists, this pre-screening step would necessarily involve a survey of local wild and domestic species, to make the database context-specific. Pre-screening has already been successfully applied to distinguish the main sources of faecal contamination in recent water and sediment studies [23, 29, 27, 31]. This pre-screening study is more complex for archaeological studies since archaeological (faunal remains, artefacts) ethnographical (written and oral history), palynological (climate and vegetation reconstruction) or geochemical (elemental and isotopic analyses) clues are not necessarily available to identify the potential species present on site during its occupation period. Including too many species in the faecal HPCP model could lead to an enhanced and misleading variance to explain and artificially create a variance noise decreasing the efficiency of species distinction. In comparison, not including a species in the fingerprint model due to a lack of context information, or for example neglecting the potential inputs of wild species, could lead to a model underestimating potential faecal source inputs to the site context and mislead results and interpretations. It is therefore crucial, when possible, to gather as much information as possible on the potential faecal inputs at a particular site to properly use faecal stanols as biomarkers to identify species from environmental samples [6, 10, 18].

However, the current model shows that the 5β-stanol fingerprints of different mammals can be clearly distinguished at the species level, even among herbivores, using the distribution of eleven faecal stanols combined with multivariate statistics. In addition, for a species with large seasonal variations in diet such as reindeer, this fingerprinting method also makes it possible to distinguish a winter-diet fingerprint from a summer diet fingerprint, when diets are more varied.

### Application and validation of the faecal fingerprint reference library to modern and archaeological sites

#### Contemporary test case: A Tofa hunting camp in the Sai͡an mountains

In the PCA model built based for the Tofa hunter camp context from the Sai͡an Mountains, omnivores (human and dogs) were clearly distinguished from herbivores (reindeer and horse) by PC1 (Fig 3A), whose main contributions came from coprostanol, 24-ethylepicoprostanol, 5β-epicampestanol and 5β-lichestanol (Fig 3B and S5 Table). As in the PCA/HCPC models including all ten species (Fig 2A–2C, S4 Table), there was a degree of overlap between the human and dog faecal fingerprints in the PCA model, probably due to the special diet of the dog D2 sample (see previous discussion) and perhaps due to the intra-species variabilities involved when fingerprinting on single individuals (see [65–67] for humans). PCA results are confirmed and further refined by the further HCPC which shows a clear distinction between omnivore and herbivore fingerprints mainly according to coprostanol, 24-ethylepicoprostanol, 5β-lichestanol, 24-ethylcoprostanol and epicoprostanol (Fig 3C, S5 Table). As discussed previously and in agreement to earlier studies ([19, 33] and references therein) the main cholesterol- and phytosterol-derived faecal stanols make it possible to distinguish diet-related fingerprint differences (omnivores versus herbivores).

Among herbivores, the species fingerprints of reindeer with contrasted diets (summer diet) are clearly separated from each other and from those of horses mainly due to 5β-lichestanol and epicoprostanol distributions (Figs 1 and 3A–3C, S5 Table). Surprisingly, 5β-lichestanol is not the main variable allowing the distinction between the faecal fingerprint of reindeer with a diet dominated by lichen (winter diet) and those with a more various diet (summer diet, S2 and S5 Tables). This discrepancy might be explained by the fact that reindeer do not follow a strict seasonal diet, but instead vary the proportion of lichens they eat in cold and warm seasons, with up to 70% of their diet being made up of lichens in the winter [68]. As lichen is most probably the main source of lichesterol for reindeer [57], the abundance of its transformation product 5β-lichestanol in reindeer faeces might not be as season-dependent as we first hypothesized.

When the concentrations of the 5β-stanols of the soil samples from the camp were compared to the PCA/HCPC models built from the reference library of dog, human, reindeer and horse faecal stanols, it was clear that none of the soil samples contained faecal signatures matching those of humans or dogs (Fig 3A–3C). One soil sample (Saian2) had a horse fingerprint, while all other soil samples had reindeer fingerprints. Significantly, the soil samples with reindeer faecal fingerprints closely matched the faecal reference samples of reindeer with a lichen-based, winter diet (Fig 3C). These results show that the PCA/HCPC models built using our eleven-stanol reference library were able to distinguish the faecal fingerprints of the two species–horses and reindeer–that frequented the part of the site from which the soil samples were taken, and also successfully identified the dominant season of use.

#### Archaeological test case: I͡Arte 6, on the I͡Amal peninsula

In the PCA/HCPC models built for the I͡Amal archaeological site, there was a clear distinction between reindeer and lemming fingerprints along PC1, whose main contributing variables were 5β-epistigmastanol, 5β-lichestanol, 24-ethylepicoprostanol, 5β-epibrassicastanol and 24-ethylcoprostanol relative abundances (Fig 4A and 4B, S6 Table). In accordance with our ten-species PCA model (Fig 2A–2C, S4 Table), omnivore (humans and dogs) and herbivore (reindeer and lemmings) fingerprints were mainly differentiated by the relative concentrations of coprostanol and 24-ethylcoprostanol along PC2. As previously discussed, there was a slight overlap between the human and dog faecal fingerprints.

Soil samples from the buried soils adjacent to the site, which were radiocarbon dated to the 5^th^-11^th^ century AD (S3 Table), were analysed for faecal fingerprints using the PCA model and corresponding HCPC generated from the relative concentrations of 5β-stanols in the human, dog, reindeer and lemming faecal reference library (Fig 4A and 4C). We found that one soil sample (Iamal4) had an omnivore fingerprint closely linked to the faecal fingerprints for dogs, and the remaining six soil samples had a winter-diet reindeer fingerprint (Iamal2, 5, 6, 7, 8 and 9). No sample presented a lemming fingerprint, which suggested the low contribution of these rodents to the background level of 5β-stanols in this area. Using the PCA model alone, one soil sample (Iamal3) appeared to contain a faecal fingerprint that was a mixture of omnivore (human or dog) and reindeer (Fig 4A). However, this apparent mixture was resolved using HCPC, which clearly showed the closer link between this sample’s faecal fingerprint and that of winter-diet reindeer (Fig 4C) and highlighted the importance of analyzing data beyond the PCA level.

The PCA/HCPC models we built using our eleven 5β-stanol reference library clearly demonstrated that reindeer eating an early spring/late autumn/winter diet predominantly based on lichen had indeed been congregating in close proximity to the human camp of I͡Arte 6 in the 5^th^-11^th^ century AD–a behaviour that would not have been exhibited by migratory wild reindeer. This result has important implications for our understanding of early reindeer domestication, and the ongoing debate about where and when this relationship developed. This case study highlights the potential of our 5β-stanol fingerprinting technique to contribute to the interpretation of human-animal relations in the past.

### Relevance of the method compared to currently-used ones

To compare the different approaches more closely, for each soil sample analysed, we compared the faecal sources determined using four stanol ratios widely used in the literature (R1, R2, R3 and R4, see Material and Methods section and S7 Table), the PCA/HCPC models built using only the relative abundances of the four 5β-stanol compounds normally used in the aforementioned ratios (coprostanol, epicoprostanol, 24-ethylcoprostanol and 24-ethylepicoprostanol), and the PCA/HCPC models built using the eleven 5β-stanol distributions used in this study.

For the Sai͡an Mountains site, the fingerprints given by the four diagnostic ratios were generally in good agreement, and identified a predominantly herbivore faecal input in soil samples (S7 Table, Fig 5). Nevertheless, for sample Saian8, the R1 ratio, which is only based on two compounds (coprostanol and 24-ethylcoprostanol), identified an omnivore fingerprint while the three other ratios identified an herbivorous one (horse = herbivore for R4 ratio [10]). The limits of this two-compound ratio has already been raised by Derrien et al. [22] when applied to the distinction between pig and cow fingerprints and is confirmed in the present study by its comparison with other diagnostic ratios. While R2 and R3 ratios gave similar herbivore fingerprints for Sai͡an soil samples, their diagnosis was different for two samples from the I͡Amal peninsula archaeological site (Iamal3 and Iamal4, S7 Table, Fig 6). As these two ratios are based on different compound distributions (coprostanol, epicoprostanol, 24-ethylcoprostanol and 24-ethylepicoprostanol for R2; coprostanol, epicoprostanol and cholestanol for R3), this discrepancy highlights the fact that diagnostic ratios are compound-dependent and that the faecal source input attributed to a soil sample is dependent on the ratio, and thus compounds, chosen.

The failure to attribute a faecal fingerprint based only on the four main faecal stanols (coprostanol, epicoprostanol, 24-ethylcoprostanol and 24-ethylepicoprostanol) can also be read into the PCA/HPCP models based on their relative abundances for both sites. For the Sai͡an Mountains site, the four-compound PCA/HPCP models do not allow a clear distinction between horse and reindeer fingerprints. Neither are dog and human fingerprints clearly separated (Figs 5 and 7, S7 and S8 Tables). Therefore this method cannot be used to identify the main faecal input sources to soil samples. The lack of distinction between dog and human fingerprints follows from the fact that the distribution of the four main compounds in their respective faeces is quite similar, as previously discussed. The same issue occurs with the four-compound PCA/HCPC model of the I͡Amal peninsula archaeological site since the human and dog fingerprints are not well separated and therefore the identification of the main faecal input source for omnivore-related soil samples cannot be done to the species level (Figs 6 and 8, S7 and S9 Tables). This methodological limitation is confirmed by the discrepancy observed between faecal source identification achieved by the four-compound PCA/HCPC models and those from ratios for 53% of our soil samples (Saian8 and Iamal3 to 9).

By contrast, the method which uses eleven compounds (PCA-11, Figs 2C, 5 and 6, S4 and S7 Tables) makes it possible to clearly distinguish the fingerprints at the species level (see above) with little overlap. As a consequence, the main faecal input sources attributed to soil samples can be achieved at the species level with more confidence than when using diagnostic ratios or four-compound PCA/HCPC models. It has to be noted that for each case-specific model, the compounds (or variables) allowing the distinction between species fingerprints are not necessarily always the same. Their respective weights will depend on the context. Thus, the five main compounds which allow the clear separation of species for the Sai͡an Mountains site are coprostanol > 24-ethylepicoprostanol > 5β-lichestanol > 24-ethylcoprostanol > epicoprostanol, followed by the six remaining compounds (Fig 3C, S5 Table). Similarly, for the I͡Amal peninsula archaeological site, these compounds are coprostanol > 24-ethylepicoprostanol > 5β-lichestanol > 24-ethylcoprostanol > 5β-stigmastanol, followed by the six remaining compounds (Fig 4C, S6 Table). If we turn to the model built from ten species fingerprints for both case-studies (Fig 2C, S4 Table), we would need three of the four main faecal stanols (coprostanol, 24-ethylepicoprostanol and 24-ethylcoprostanol) to distinguish between omnivore and herbivore fingerprints. Despite the fact that species determination can be done in some cases with a small set of 5β-stanols does not mean that the other compounds are superfluous. As demonstrated by the case of trying to distinguish between dogs and humans, stanol fingerprinting is much more successful when employing eleven compounds instead of four. Part of this success is due to the fact that these two case studies structured to an unusual degree around 5β-lichestanol, which is the stanol present in higher proportions in reindeer faeces than that of other species (Fig 1). If we were to move to a non-Arctic case study, we would anticipate that a different set of compounds would play a greater role in the building of HCPC clusters and identification of specific species.

These results also highlight the importance of using appropriate statistical techniques and bundles of compounds to improve species fingerprint distinction. Indeed, PCA analysis was first used by Leeming et al. [19] to distinguish the main trends among steroidal compounds to identify specific fingerprints. However, they did not only use 5β-stanols but also sterols and 5α-stanols, which can naturally occur in the environment (S2 Table and [10]) and stanones, which are intermediate products in the transformation process of sterols into 5β- and 5α-stanols [19]. As a consequence, their PCA model was not based only on pure faecal biomarkers, which could lead to bias when applied to the interpretation of environmental samples. Nevertheless, their pioneering study could also have been improved by using a further hierarchical analysis (canonical, HCPC etc.) following their PCA as PCA distinction is only visually-based on two PCs while hierarchical analyses use more dimensions and therefore take into account more variance observed between samples. Shah et al. [4] also used non faecal steroids in their study (5α-stanols and sterols in addition to the faecal 5β-stanols) in combination with canonical analysis and were not able to distinguish between herbivore species. It might have been possible if only using 5β-stanols. These two previous studies focused on several and diverse species, which also makes it more complex to distinguish species’ fingerprints, since there is an increased variance to explain. When focusing on a smaller number of species with contrasted steroid distributions (e.g. human, cattle and pig), it is however possible to distinguish between species fingerprints using both 5β- and 5α-stanols and to successfully apply it to water samples [22–24, 27, 28, 31] without using further hierarchical analysis after PCA. Importantly, the occurrence of 5α-stanols in large amounts in soils, especially sitostanol (S2 Table), and their lack of specificity as faecal biomarkers make them irrelevant for soil-related studies.

Finally, in contrast to the analysis of other faecal lipid biomarkers like bile acids (e.g. [3, 11]), the analysis and quantification of the eleven 5β-stanols considered here can be achieved on a single GC-MS injection, since these compounds are part of the same fraction. As a consequence, it is neither more expensive nor more time consuming to improve the accuracy of faecal fingerprinting by switching from four to eleven compounds when only considering 5β-stanols.

## Conclusions

Our results call into question the validity of using simple ratios and even multivariate statistics based on only four 5β-stanols for species-specific faecal fingerprinting. The faecal fingerprint reference library used here, and the use of PCA and HCPC models built using eleven 5β-stanols, provide much more precise faecal source attributions. The fingerprinting method employed here overcomes the limitations of using simple ratios involving only four 5β-stanol compounds (coprostanol and epicoprostanol, 24-ethylcoprostanol and 24-ethylepicoprostanol) to determine the main sources of faecal inputs in environmental and archaeological samples. For the two cases studied here, the model used confirmed the past presence of horses and reindeer on the Sai͡an Mountains site and reindeer and dogs on the I͡Amal peninsula archaeological site.

As more faecal reference samples from a larger number of mammalian species consuming different diets are added to our faecal reference library, species identification and the breadth of its applications will continue to improve.

## Supporting information

S1 Fig Example GC-MS chromatogram (total ion current) from a reindeer sample (R12, see S2 Table). Retention times correspond to the analytical method used for this sample as described in Materials and Methods and S2 Table. Trivial names of the eleven 5β-stanols considered in this study are labelled black (S1 Table). Trivial names of 5α-stanols and sterol precursors are labelled grey.
(TIF)

S2 Fig Mass spectra and summarised fragmentation patterns of TMSi ether derivatives of eleven 5β-stanols found in faecal samples. M+ = molecular fragment. SC = side chain. TMSiOH = trimethylsilanol fragment. Me = methyl. Identification of 5β-lichestanol was made by comparison with the mass spectra of the TMSi ether derivative of stellasterol (24-methyl-5α-cholesta-7,22E-dien-3β-ol), which is structurally similar except for the B-ring double bond location [69]. Identification of both 5β-brassicastanol and 5β-epibrassicastanol was made by comparison with the mass spectra of the TMSi ether derivative of the 5α-brassicastanol (24-methyl-5α-cholest-22E-en-3β-ol, [70]). The mass spectra of the TMSi ether derivative of 5β-epibrassicastanol is similar to the one of 5β-brassicastanol, therefore we did not present it here.
(TIF)

S1 Table 5β-stanol names and GC-MS properties. 5β-stanols considered in this study and common 5α-stanols and sterols found in samples analyzed. Chromatographic and mass-spectrometric properties for identification and quantification of listed compounds are also presented.
(XLSX)

S2 Table Sample information and 5β-stanol distribution. Information relative to sample collection and analytical method as presented in Materials and Methods, 5β-stanol distribution and sum, concentration of main 5α-stanols and recovery efficiency of internal standard when added.
(XLSX)

S3 Table AMS dating information for the buried soils at the I͡Arte 6 site, I͡Amal peninsula. Red text indicates outliers. The radiocarbon calibration multiplot provides a graphical summary of the data in the S2 Table. The results show that the buried soils adjacent to I͡Arte 6, which contained faecal lipids, developed between the 6^th^ and early 11^th^ century AD.
(XLSX)

S4 Table Characteristics of the first four principal components, variables and species distinction of the PCA built on the distribution of 5β-stanols in all ten species faeces. **Characteristics of the HCPC model built on the PCA.** Variables and species loadings on PCs 1, 2, 3 and 4 show which variables contribute the most (absolute value) to distinguish between species as represented by “General distinction” regression coefficient and related relevant significantly distinguished species. For each PC, the more two species have a high loading difference (relative value) the more they are distinguished by this PC. For the HCPC model, the main variables (or compounds) explaining the hierarchical cluster tree building have the highest absolute v.test/Etat2 values. The species fingerprints gathered within the main clusters are identified and the main variables responsible for the distinction between main clusters are given by the highest v.test values.
(XLSX)

S5 Table Characteristics of the first four principal components, variables and species distinction of the PCA built on the distribution of 5β-stanols in human, dog, horse and reindeer faeces (selected for the Tofa site context, Sai͡an Mountains). **Characteristics of the HCPC model built on the PCA.** Variables and species loadings on PCs 1, 2, 3 and 4 show which variables contribute the most (absolute value) to distinguish between species as represented by “General distinction” regression coefficient and related relevant significantly distinguished species. For each PC, the more two species have a high loading difference (relative value) the more they are distinguished by this PC. For the HCPC model, the main variables (or compounds) explaining the hierarchical cluster tree building have the highest absolute v.test/Etat2 values. The species fingerprints gathered within the main clusters are identified and the percentage of the species samples are given (%). The main variables responsible for the distinction between main clusters are given by the highest v.test values.
(XLSX)

S6 Table Characteristics of the first four principal components, variables and species distinction of the PCA built on the distribution of 5β-stanols in human, dog, horse and reindeer faeces (selected for the I͡Amal site context). **Characteristics of the HCPC model built on the PCA.**Variables and species loadings on PCs 1, 2, 3 and 4 show which variables contribute the most (absolute value) to distinguish between species as represented by “General distinction” regression coefficient and related relevant significantly distinguished species. For each PC, the more two species have a high loading difference (relative value) the more they are distinguished by this PC. For the HCPC model, the main variables (or compounds) explaining the hierarchical cluster tree building have the highest absolute v.test/Etat2 values. The species fingerprints gathered within the main clusters are identified and the percentage of the species samples are given (%). The main variables responsible for the distinction between main clusters are given by the highest v.test values.
(XLSX)

S7 Table Comparison of diet and species identification between ratios and multivariate analyses for both case study sites. (XLSX)

S8 Table Characteristics of the first four principal components, variables and species distinction of the PCA built on the distribution of the four main 5β-stanols (coprostanol, epicoprostanol, 24-ethylcoprostanol and 24-ethylepicoprostanol) in human, dog, horse and reindeer faeces (selected for the Tofa site context, Sai͡an Mountains). **Characteristics of the HCPC model built on the PCA.** Variables and species loadings on PCs 1, 2, 3 and 4 show which variables contribute the most (absolute value) to distinguish between species as represented by “General distinction” regression coefficient and related relevant significantly distinguished species. For each PC, the more two species have a high loading difference (relative value) the more they are distinguished by this PC. For the HCPC model, the main variables (or compounds) explaining the hierarchical cluster tree building have the highest absolute v.test/Etat2 values. The species fingerprints gathered within the main clusters are identified and the percentage of the species samples are given (%). The main variables responsible for the distinction between main clusters are given by the highest v.test values.
(XLSX)

S9 Table Characteristics of the first four principal components, variables and species distinction of the PCA built on the distribution of the four main 5β-stanols (coprostanol, epicoprostanol, 24-ethylcoprostanol and 24-ethylepicoprostanol) 5β-stanols in human, dog, horse and reindeer faeces (selected for the I͡Amal site context). **Characteristics of the HCPC model built on the PCA.** Variables and species loadings on PCs 1, 2, 3 and 4 show which variables contribute the most (absolute value) to distinguish between species as represented by “General distinction” regression coefficient and related relevant significantly distinguished species. For each PC, the more two species have a high loading difference (relative value) the more they are distinguished by this PC. For the HCPC model, the main variables (or compounds) explaining the hierarchical cluster tree building have the highest absolute v.test/Etat2 values. The species fingerprints gathered within the main clusters are identified and the percentage of the species samples are given (%). The main variables responsible for the distinction between main clusters are given by the highest v.test values.
(XLSX)
