# Supplementary figures and images for: Faecal biomarkers can distinguish specific mammalian species in modern and past environments
Source: PLoS One. 2019 Feb 7;14(2):e0211119. doi: 10.1371/journal.pone.0211119 (PMC6366745; doi:10.1371/journal.pone.0211119)

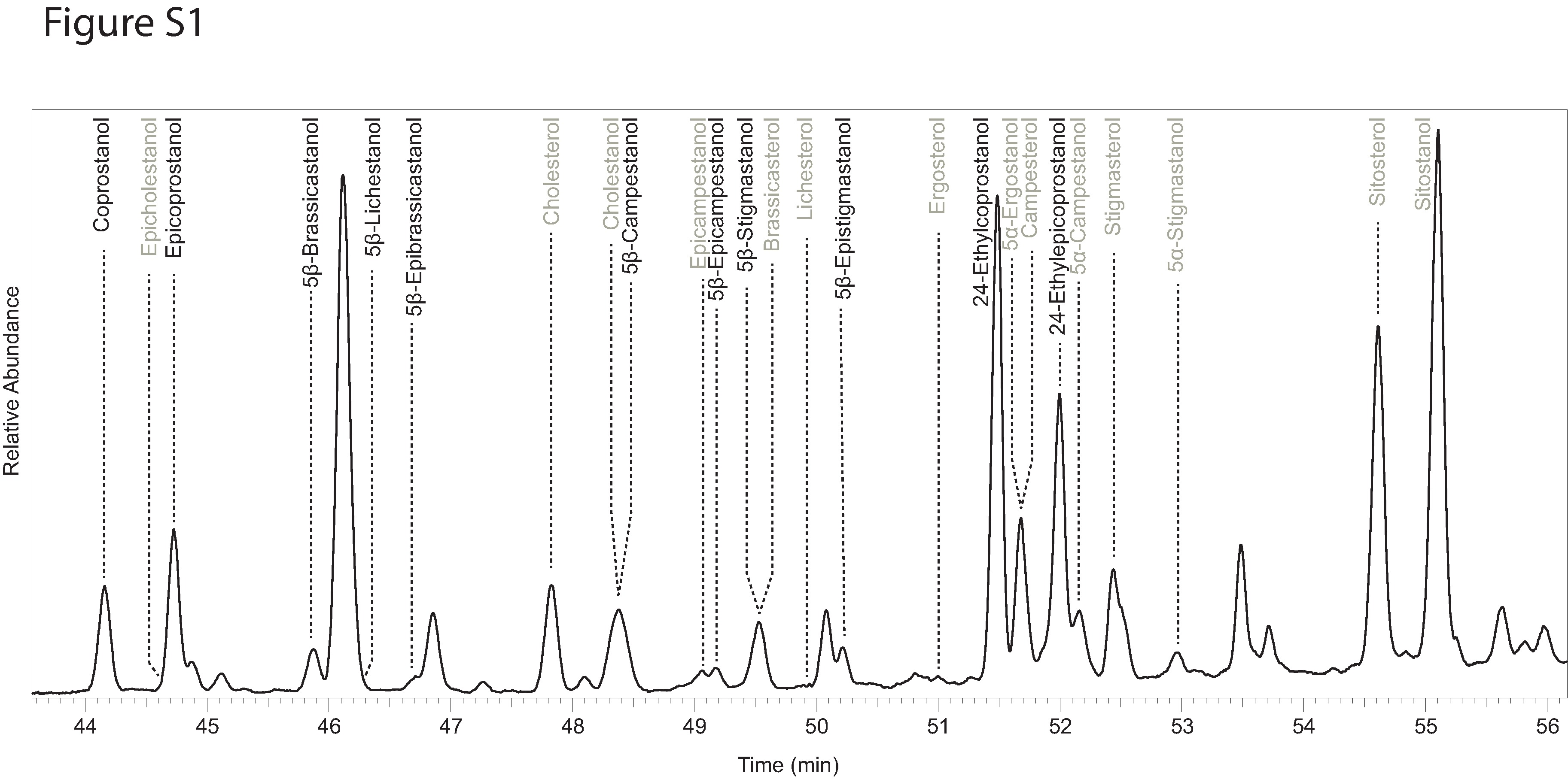

Supplement: S1 Fig — Retention times correspond to the analytical method used for this sample as described in Materials and Methods and S2 Table. Trivial names of the eleven 5β-stanols considered in this study are labelled black (S1 Table). Trivial names of 5α-stanols and sterol precursors are labelled grey. (TIF) [file pone.0211119.s001.tif]

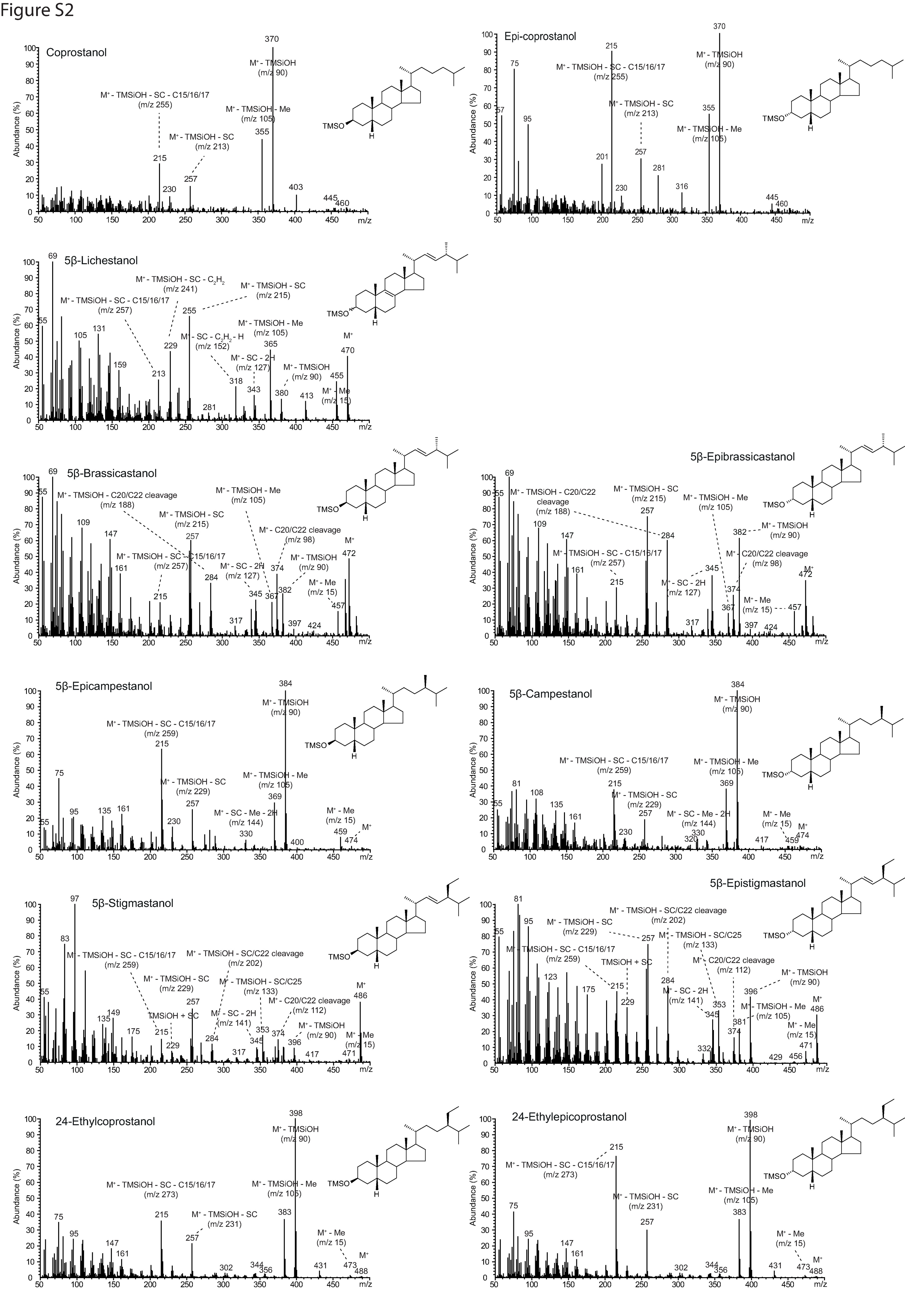

Supplement: S2 Fig — M+ = molecular fragment. SC = side chain. TMSiOH = trimethylsilanol fragment. Me = methyl. Identification of 5β-lichestanol was made by comparison with the mass spectra of the TMSi ether derivative of stellasterol (24-methyl-5α-cholesta-7,22E-dien-3β-ol), which is structurally similar except for the B-ring double bond location [69]. Identification of both 5β-brassicastanol and 5β-epibrassicastanol was made by comparison with the mass spectra of the TMSi ether derivative of the 5α-brassicastanol (24-methyl-5α-cholest-22E-en-3β-ol, [70]). The mass spectra of the TMSi ether derivative of 5β-epibrassicastanol is similar to the one of 5β-brassicastanol, therefore we did not present it here. (TIF) [file pone.0211119.s002.tif]
